# From theory to practice in implementation science: qualitative insights from the implementation model developed by a commercial eMental Health provider

**DOI:** 10.1186/s43058-024-00610-y

**Published:** 2024-07-04

**Authors:** Sofia Bastoni, Charlotte Marijne van Lotringen, Hanneke Kip, Robbert Sanderman, Lisette J. E. W. C. van Gemert-Pijnen, Anne van Dongen

**Affiliations:** 1 Centre for eHealth and Wellbeing Research, Department of Psychology, Health and Technology, University of Twente, Enschede, Netherlands; 2 Department of Research, Transfore, Deventer, Netherlands; 3 Department of Health Psychology, University Medical Center Groningen, University of Groningen, 9712 Groningen, The Netherlands

**Keywords:** eHealth, eMental health interventions, Implementation frameworks, Stakeholder involvement, CFIR

## Abstract

**Background:**

Although eMental health interventions are a viable solution to address disparities in access to mental healthcare and increase its efficiency, they still face challenges of implementation. Literature highlights numerous barriers such as diffusion of responsibility and unclear expectations of what implementation entails might hinder this process. While research mostly focuses on analyzing these barriers, there is an urgent need to increase uptake in practice. In turn, commercial companies focus mostly on increasing uptake, while overlooking research outputs. To bridge the gap between research and practice, attention to how implementation occurs in practice is required. This study investigates “Make it Happen” (MiH), the implementation model developed by the eMental Health company Minddistrict, aiming to gain more insight into operationalizing implementation frameworks by 1) describing MiH and its conceptual underpinnings, and 2) gaining lessons learned from the development of MiH. Ultimately, this work aims at improving existing scientific frameworks by extending them with knowledge from practice.

**Methods:**

First, individual interviews and focus groups with Minddistrict implementation managers were performed. Second, individual interviews with project leads in mental healthcare organizations that were involved in the implementation of Minddistrict were conducted. Within Minddistrict, 7 implementation managers and account managers were involved, in addition to 11 project leads from mental healthcare organizations. Data were elaborated with thematic analysis.

**Results:**

A comprehensive description of MiH and its 5 main phases was achieved. During the 1) Onboarding phase, implementing organizations are guided by Minddistrict to build a team responsible for implementation, which then 2) designs patient and client journeys, 3) builds, tailors and configures their offer, 4) trains key-users and, 5) evaluates the success of implementation. All participants had extensive and aligned definitions and articulated expectations on implementation. Points of improvement for the model such as role ambiguity and excessive workload were identified. As strengths, internal motivation and good relationships with the provider were valued.

**Conclusion:**

The present study highlights the importance of clear role division and stakeholder engagement in implementation processes, and suggest that a strong collaboration between companies and academia could optimize implementation efforts and ensure a better fit between humans, context, and technologies.

**Supplementary Information:**

The online version contains supplementary material available at 10.1186/s43058-024-00610-y.

Contributions to the literature

- eMental health technologies are a promising solution to address (among others) access barriers to mental health care, But they are often not properly implemented and end up abandoned or misused.
- Although many implementation guidelines exist in academic literature, they are too abstract to be applied in practice. On the other hand, eMental health solutions are also developed outside of academia, often lacking a strong theoretical foundation.
- Our findings pave the way to improve the collaboration between research and practice. We describe an implementation model developed by a commercial eMental health provider and propose an integration example for designing and implementing eMental health technologies.

## Background

Mental disorders make up more than 25% of non-fatal disease burden worldwide [1] leading to all sorts of individual consequences, such as reduction of the quality of life [2]. Despite these alarming statistics, addressing mental health issues is hindered by limited access to mental healthcare. One major contributing factor is the shortage of mental healthcare professionals, a concern prevalent in both low-income and high-income countries [1, 3]. Social commitments, such as providing care to a loved one, can put an even greater strain on mental health. In fact, several studies highlight the negative impact of informal care on mental health in Dutch caregivers [4, 5]. The Dutch Association of Mental Health and Addiction Care [3] anticipates a shortage of 14,000 mental health care workers in the Netherlands by 2032. Hence, there is an urgent need for innovations that can support people with mental illness in dealing with their disorder, without resulting in an additional burden on a healthcare system that is already under pressure. eMental health, defined as "mental health services and information delivered or enhanced through the Internet and related technologies" [6], is widely acknowledged as a promising solution to tackle access challenges [7, 8]. eMental health also offers numerous advantages, including cost reduction and the flexibility to tailor services to individual needs while maintaining standardized quality [6]. Furthermore, the (cost)effectiveness of eMental health technologies such as internet-based interventions has been observed in several contexts, for example in depression and anxiety disorders treatment [9, 10] or cognitive behavioral therapy settings [11].

While its potential is widely recognized, eMental health still faces challenges of implementation [12]. In the context of eHealth and eMental health, Implementation can be defined as a set of “concrete activities taken to make patients and healthcare providers start and maintain use of new evidence within the clinical setting” [13]. The process of adoption of a technology, from the organizational decision of taking up a new system, to the actual integration of the innovation in every day practices, is a multifaced and complex process [14, 15], that entails numerous potential barriers. Examples of prominent barriers are uncertainty and diffusion of responsibility for implementation tasks [16], misconceptions or partial mental models of implementation within the actors involved [17] and the complexity of implementation itself as a process [14, 15]. While numerous implementation frameworks and theories are available in academic literature [12, 14, 18–21], navigating the complexity and choosing the right one is usually a strenuous task [22]. Moreover, a study by Birken and colleagues [23] highlights implementation frameworks are often misused, underused, or superficially used. As most implementation studies currently focus on identifying barriers and facilitators [24, 25], insight on how to make the best use of available frameworks is still needed. Additionally, as far as we are aware, this paper offers a unique perspective by documenting the development of an implementation model originating outside of academia.

To gain insight on how implementation guidelines are developed and applied in commercial contexts and to explore how academia and market settings can learn from each other in the realm of implementing eHealth, the current study takes a closer look at the implementation model developed by Minddistrict, "Make it Happen" (MiH), and its conceptual underpinnings. To do so, the experience and advice of the two most relevant stakeholder groups (i.e., from both the commercial and healthcare sides) involved with the implementation of Minddistrict and MiH are explored.

Practically, this study aims to address two goals:

1. Describing “Make it Happen”, the implementation model developed by the commercial eMental Health provider Minddistrict.
2. Map the experiences of people who were involved in the implementation of Minddistrict MiH from a commercial and healthcare perspective, to gain lessons learned from the points of improvement and strengths of this model.

## Methods

The present study is a thorough description and a qualitative evaluation of MiH, Minddistrict’ s implementation model, achieved with a multi-method iterative research approach [1]. Research questions and research phases were developed iteratively and collaboratively with Minddistrict. Table 1 provides an overview of the phases, aims, methodologies, and participants. This study is reported in accordance with the APA Qualitative Journal Article Reporting Standards (JARS-Qual) [26].

**Table 1:** Overview of phases, aims, methodologies, and participants

Phase	Aim	Method	Population	N	Duration (mins)
1	Description of MiH & definition of implementation	Semi-structured 1:1 interviews	Implementation Managers	6	60
	Description of MiH & definition of implementation	Semi-structured 1:1 interviews	Account Managers	1	60
	Description of MiH	Focus group	Implementation Managers	6	90
	Description of MiH	Desk research	N/A	N/A	N/A
	Description of MiH	Visualized overview	N/A	N/A	N/A
2	Qualitative appraisal of MiH- Identification of stakeholders	Discussion	Implementation Managers	2	60
	Qualitative appraisal of MiH	Semi-structured 1:1 interviews	Project Managers	10	60
	Qualitative appraisal of MiH	Semi-structured 1:1 interviews	Key user	1	60

### Setting

Minddistrict is a Business-to-Business eHealth company, providing an online platform hosting modules for mental health. Specifically, they deliver app-based online interventions focusing on a broad range of topics such as anxiety, mindfulness, lifestyle, ADHD, etc. Their clients, mental healthcare organizations, can acquire the use of the platform to be able to offer digitally enabled and blended therapy to their patients. These healthcare organizations are guided in the implementation process by the implementation managers, a team of 6 Minddistrict employees with different backgrounds, whose main task is to ensure the MiH is applied and followed to guide the healthcare organizations throughout the implementation process. Within mental healthcare organizations, a group of employees from different departments is appointed responsible for the implementation of Minddistrict. These groups are led by a project manager (hereby referred as: project lead). Within the implementing organizations, therapists (or other care professionals, referred by Minddistrict as “Key Users”) use Minddistrict’s modules with their patients.

### Phase 1- Description of the MiH model

**Fig. 1:**
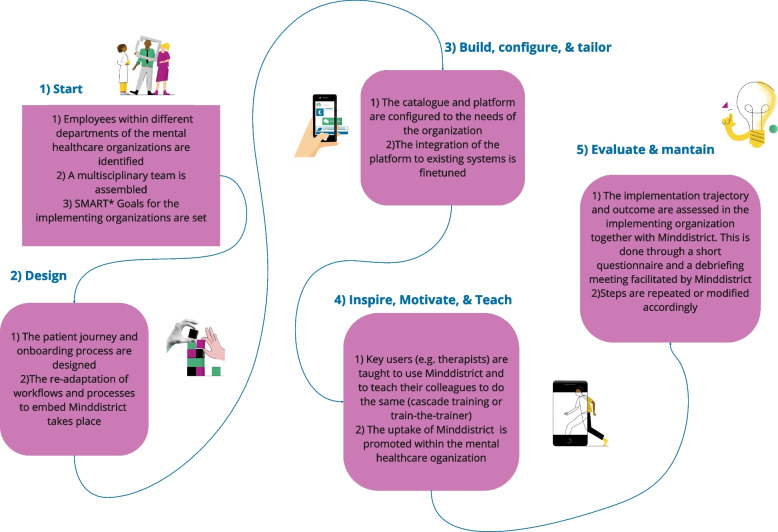
Phases of Make it Happen (MiH) and main activities within the phases. *SMART (Specific, Measurable, Achievable, Relevant, and Time-bound) goal setting is a commonly used framework and best practice in project management, often credited to the work of George T. Doran [28]

Phase 1 aimed at describing MiH, its conceptual foundation, and its functional and procedural elements. To achieve a comprehensive understanding of MiH, a variety of methods were employed: First, i) one-to-one semi-structured interviews with implementation and account managers in Minddistrict (N = 7), and ii) a follow up focus group with implementation managers took place. Parallelly, iii) desk research on internal documents (e.g., introduction slides for their clients; files describing procedures and definitions; task-management platforms etc.) was conducted. Additional file 1 illustrates the interview and focus group guides and the focus group visual boards. The interview guide inquired implementation and account managers’ definition of implementation, including its steps, stakeholders involved and tasks. The focus group guide inquired 1) the main phases of the MiH and activities within the phases the origins of the method, and 2) follow up questions based on the Consolidated Framework for Implementation Research (CFIR) [18], to make sure main information across the CFIR domains (intervention, outer setting, inner setting, individual and process) was obtained. Focus group prompts organized according to the CFIR domains are available in Additional File 1. The interviews took place online or in person according to the availability of the interviewees, by two researchers (SB and CmVL). The focus group was conducted online and the Miro (www.miro.com) board was used as a visual aid for participants during the focus groups. Interviews and focus groups were transcribed verbatim. Data from the individual interviews were analyzed inductively by one researcher (SB) with thematic analysis [27], focus group data was analyzed by one researcher and checked by another researcher (CMvL). A visualized overview of the model and the different phases was created based on this outcome and verified with Minddistrict implementation managers (Fig. 1).

### Phase 2- Qualitative appraisal of the MiH

A list of possible stakeholder groups (namely: Therapists and other key-users, Project leads, IT employees and support staff) who had experience with the most updated version of the MiH i) was proposed by the research team and discussed with the implementation team. Two main stakeholder groups, project leads and therapists (and other key users), were selected because they ii) they had a birds eye perspective on the implementation model and iii) they participated in (most of) the implementation sessions (since mid-2022). Recruitment was stopped when all project managers and therapists matching inclusion criteria (i, ii, iii) were approached. The interview guide (Additional file 1) was developed by the research team and discussed with Minddistrict’ s implementation managers, also identifying relevant themes that emerged during phase 1. The interview guide inquired general memories about the implementation process, and strengths and points for improvement for the model in each phase. The interviews took place online between June and August 2023. Most of the interviewees were conducted in English language, two interviews were conducted in Dutch and then translated. When a member check or clarifications were necessary, stakeholders were invited for a second round of interview, and in every case encouraged to keep communication (via email or preferred mode) with the research team in case they wanted to add more information at a later stage. Data were inductively coded by one researcher (SB) and partially reviewed (10%) by a second researcher (CMvL). The percent agreement between the two raters was 85.71%, suggesting a high level of consistency. Conflicts were solved through discussion. Recurring themes and subthemes were identified and discussed by the two researchers (SB and CMvL) until consensus on the coding scheme was reached. Thematic analysis was performed according to Braun and Clarke’s guidelines (2006), first by familiarizing with the data, then identifying an initial synthesis of recurring themes. Subthemes were then identified.

#### Application of the findings

Once data were analyzed and elaborated, a participatory feedback session was conducted together with Minddistrict’s implementation team. The aim of this session was twofold, first to conduct a member check on obtained results, and secondly to brainstorm on how to apply possible improvements identified within the context of the present. Reporting is beyond the scope of the present work.

## Results

### Phase-1 Description of Make it Happen (MiH)

The Make it Happen (MiH) model was developed by the Minddistrict implementation team (the last version, that is object of the present study, was finished in 2022) with the goal of systematizing, improving and guiding implementation efforts in implementing organizations. Figure 1 summarizes the results of the focus group, illustrating the five phases of MiH and the main activities within those phases.

#### Definitions of Implementation

To understand if implementation managers and project leads in mental healthcare organizations had aligned definitions of implementation (and therefore aligned expectations of what implementation entails), both groups were asked about their definitions of implementation during individual interviews. Table 2 provides quotes and other relevant keywords from the interviews.

**Table 2:** Definitions of implementation in implementation managers of Minddistrict and project leads in mental healthcare organizations

Definitions of implementation					
Implementation managers in Minddistrict					
Implementation Manager 1	Implementation Manager 2	Implementation Manager 3	Implementation Manager 4	Implementation Manager 5	Implementation Manager 6
Implementation is making things work in practice Other key elements: flow; alignment in goals; sensemaking process	Implementation is behavior change, both in short and long term, both personal and organizational levels Other key elements: digital, interaction, future, growth integration	Implementation is turning an abstract idea into reality Other key elements: putting a decision or plan into effect; execution; behavior change	Implementation is an organization or entity wanting to start using something new; making sure that the product is integrated well into the work procedures and processes of this organization Other key elements: promoting agency and motivation	Implementation means ensuring adoption Other key elements: Exploring motivations to use, setting goals; motivating and inspiring the right people; evaluating	Implementation is starting to use something new
Project leads in the implementing organizations					
Project lead 1	Project lead 2		Project lead 3 e 4	Project lead 5	Project lead 6
Implementation is the entire process [mentions the phases of the MiH] from beginning to end Key elements: kickoff, forming a group, sessions, technical aspects trainings	Implementation is something that needs to go smoothly with everyone, where the outcome is as envisioned beforehand Other key elements: Importance of knowing their own tasks, responsibilities, and time to allocate to them Other key words: introduction of new things, change, difficulties, underestimation of workload		Implementing eHealth solutions means having a clear vision and goals. The innovation needs to be an integral part the treatment processes and daily workflow Other keywords: Technical implementation (related to the use of software; trainings). Stakeholders: high management needs to support it, healthcare side, content people, support people (IT)	Implementation starts with a commission, but then continuous implementation and maintenance are necessary to guarantee success Other key elements: High management needs to be on board; processes need to be integrated	Implementation has two phases: i) learning how something works technically and familiarizing and ii) really incorporating it into everyday practices
Project lead 7	Project lead 8		Project lead 9	Project lead 10	Project lead 11
Implementation is familiarizing with the innovation at an organization level	Implementation starts with identifying a need (in this case a system like Minddistrict was the need). Then all the steps follow (refers to implementation model) Other key elements: Implementation is shared responsibility		The interviewee was not familiar with implementation before this project	Implementation means getting people motivated Other key elements: Continuous implementation and ambassadorship through innovators	Implementation (of Minddistrict) is creating a patient journey and constant evaluation and maintenance accordingly

##### Definitions of Implementation within Minddistrict

Implementation managers of Minddistrict were not able to trace the model or their definition of implementation in implementation science literature, but they showed a quite aligned definition of implementation with each other. They often referred to the phases of MiH to describe what implementation is to them, and they produced answers quickly. Their definitions of implementation can be synthesized as: *“the systematic process of turning projects into something concrete, in this case by initiating the use of an innovation within the organization”.* According to them, implementation also entails successfully integrating the innovations into existing workflows and procedures, ensuring their adoption. This process requieres achieving behavior change, both in the short and long term, at both individual and organizational levels. Key elements include promoting a common vision, facilitating a smooth flow of information and processes, and fostering a sense of agency and motivation among stakeholders.

##### Definitions of Implementation within mental healthcare organizations

Project leads of mental healthcare organizations also had a rather articulated and multi-level definition of implementation. In most cases implementation was defined as “*a complex, full spectrum, and articulated process, that encompasses all efforts to apply an innovation, such as a new technology, in everyday practice*”. Crucial elements of this process according to the participants are bringing awareness of the innovation through the organization; inspiring and motivating people involved in the uptake; developing clear goals and a vision for the use of the innovation; taking care of the more technical aspects of implementation and finally evaluating and taking care of the continuation of the project throughout time. Furthermore, the process involves several stakeholders: starting from the providers (in this case Minddistrict), people in high management, who are responsible for the decision-making aspects, people in the healthcare side, taking care of content elements, employees who have a supporting function in IT (Information Technology), taking care of the practical aspects.

### Phase 2- Qualitative appraisal of the Implementation model

#### Sample characteristics

The implementing organizations included had different focuses, e.g., mental health of adults, children and adolescents, nursing homes, depression, psychiatric disorders, addiction, and smoking cessation. Eleven professionals from ten organizations were interviewed, ten of them were project managers in the implementing organizations and one of them was a key user (dietician). None of the therapists who matched the inclusion criteria were able to participate in the interviews.

#### Strengths, weaknesses, and potential improvements of MiH

Thematic analysis revealed three main overarching themes in the appraisal of MiH: strengths, weaknesses, and related potential improvements suggested by the interviewees. Within these overarching themes, we identified recurring sub-themes, which we delve into in the following paragraphs. Additional file 2 provides an overview of themes and subthemes and number of times they were mentioned.

##### Strengths

One prominent theme, strengths, emerged frequently (coded 44 times). Within that theme, recurring elements were identified.

For example, project managers in the implementing organizations, appreciated their strong relationships with implementation managers, who were described as available and knowledgeable. Specifically, they appreciated being able to rely on the implementation managers’ “substantive knowledge” support on content-specific matters, and in general valued the multidisciplinary teams that were built during the implementation trajectory (comprising the implementation managers and employees from different departments within the mental healthcare organizations).

Also, overall organization of the tasks was judged as clear and the way that the various tasks to do were illustrated was efficient. More specifically, most interviewees found it useful to have an overview of tasks planned for the implementation trajectory, with deadlines and key-persons responsible for each task. To do so, Minddistrict chose to use the www.monday.com platform, a collaborative project management tool in which they would store an overview of tasks and deadlines.

Motivation emerged as another key factor in successful implementation. Implementation managers from Minddistrict were generally considered as motivated, but project managers reported different levels of motivations *within* their own organizations. Especially, organizations in which the key actors were more motivated, usually found it a facilitator for implementation. Not only would they work better if they were motivated, but they found it better when the motivation was intrinsic (as opposed for example to an external drive to take up Minddistrict due to financial incentives from the government). Lastly, fostering motivation in actors involved was mentioned as a potential mitigator for (some) implementation issues.

Another strength from our part, our contribution, was that we really want this platform and this kind of platforms. It didn’t come from a manager saying we must use it;it came from ourselves.—Interviewee 9.

Another element that was considered important in implementation was goal setting. In fact, the entire goal-setting phase was often described as challenging but overall valuable for project managers. During this phase, the implementation managers of Minddistrict guide the team in the implementing organizations into settings goals to reach for their implementation trajectory, for example in terms of metrics to reach. Participants who had previous experience with goal-setting practices, reported it was beneficial for them to already know the process and usually found it smoother. Knowing why a goal is relevant was also reported as a facilitator to setting clear goals from one interviewee. Specifically, starting with broad, vision-like goals (e.g., expanding access to mental healthcare) and later operationalizing them (e.g., reaching X number of new patients in a certain time point) was seen as a helpful approach to relate to their goals without treating them as sheer “KPIs” (Key Performance Indicators) or targets. On the other hand, project managers in organizations that were not familiar with goal setting practices often reported setting vague goals or in general giving little importance to goals afterwards (e.g., in the evaluation phases).

The platform training, using a "train-the-trainers" format was well-received by all interviewees. In fact, training would take place mostly in real life, but online trainings were also possible. Key users of Minddistrict would be trained, using a training platform with the same look and feel of Minddistrict. In that module, they were able to onboard “practice” clients, and chose the modules they would like to give them. Key users who were trained, were also trained to train their colleagues later. This modality is also known as “cascade training” [29]. Familiarizing with the platform was generally evaluated as an unproblematic step, in fact most interviewees describe the training process as “smooth”, even in online settings. As a potential improvement, sub-group training tailored to specific departments within organizations was suggested. This way, examples and other elements of the trainings could be tailored to the specific context of care (e.g., eating disorders vs anxiety disorders vs depression etc.), and therefore be more relatable for the end users.

The possibility to customize the platform to the organizations’ needs and characteristics was valued by 4 participants. Most often, that translated in the creation of tailored welcome modules for patients to be introduced to the organizations and their care goals. To help with the customization process, interviewee 11 emphasizes the importance of real-life examples in shaping a patient's journey.

“We will just go through the entire patient journey, and they [hypothetical patient journeys] were really practical. It was really obvious, and you didn't have to imagine many things because there were a lot of real examples that we used. Colleagues that were in the group came up with “Remember, patient X who had this and that?” – Interviewee 11.

Apart from customizing the welcome module and tailoring the choice of modules available, organizations adopting Minddistrict have the possibility to build their own modules from scratch (for example to offer support to a specific target group or address specific issues that cannot be addressed optimally using existing modules). Although this activity is generally an appreciated perk in theory, most interviewees reported being very interested but not having time or resources to do it. In other words, the great amount of energy, knowledge and time investment that goes into this activity is often considered too much. Only one of the interviewees reported their organization successfully built their own module. As a potential improvement, other interviewees manifested their interest in being able to adopt modules developed by similar organizations. This way, they would be able to access more specific content (e.g., for a specific patient population) without having to put in the effort to build one from scratch.

##### Barriers

On the other hand, weaknesses (coded 77 times) of MiH were also identified. A significant challenge identified by all interviewees was the underestimated workload, which is often related to an insufficient time allocated to the implementation process. Participants noted that there are different levels of implementation, with initial awareness and education preceding a deeper integration into daily practice. The time required for this deep integration was often underestimated.

As an example, interviewee 7 says:

“And that was nice that it was easy to to go with, but there was more work to do than we initially thought because it it was very hard to imagine how many hours work it was going to be.”

Tight time schedules are closely tied to the management of expectations, another recurring theme among the barriers. For example, ambiguity within the platform's structure, tasks, and roles also emerged as a significant challenge for project leads, contributing to a diffusion of responsibility. Clear expectations about role division and tasks were recognized as a vital factor in preventing disappointment and frustration. Communication challenges were mentioned, particularly when implementation managers were unavailable for extended periods due to illness.

“In my view, implementation is something that you want to go smoothly with everyone, where the outcome is as you envisioned it beforehand. Knowing that when you introduce new things, it brings about change, and that can sometimes be difficult for employees. So, I think it's very important, and that's maybe something we underestimated beforehand, to know what your tasks and responsibilities are, and the time investment required, so that you can allocate it properly and not find yourself having to rearrange things during the process, which is not pleasant for anyone at that moment.” – Interviewee 1.

The overwhelming choice of modules available on the Minddistrict platform presented another challenge. As potential improvements, additional filter options and recommended module lists to reduce decision-making pressure were indicated. Identified practical barriers included inadequate infrastructure, accessibility issues, and integration with existing platforms.

“If a client doesn't have an Internet connection yet, we are stuck, and we cannot do anything”- Interviewee 10.

Finally, critical aspects related to heterogeneity were identified. In fact, multiple interviewees reported their organization differed from the standard of mental healthcare organizations in the Netherlands, for example by being smaller in size or having different target groups. In this case, interviewees reported that it was necessary for them to deviate from the standard steps of the implementation model to accommodate their peculiarities. Particularly, in one case patients were led in the use of Minddistrict by social workers (an unprecedented key-user group in Minddistrict) rather than therapists. The project lead in this organization reported that preparatory work was needed to help the social workers in their new role, and some flexibility was required from Minddistrict’ s implementation managers to adapt to this different target group of key-users. Moreover, interviewees reported to have experienced a heterogeneous reaction to the introduction of the innovation. While some colleagues were more enthusiastic and motivated, others were described as “resistant” or “laggards''. Furthermore, there are different perspectives within an organization as the top management or the project leads who usually initiate the integration of new technologies consider them as a top priority. Key users such as therapists on the other way, might perceive innovation as a top-down obligation disrupting their daily practice.

When asked about possible ways to address these issues, several interviewees hinted at some sort of ambassadorship mechanisms. In other words, having individuals positively advocating for the uptake of the innovation, preferably within the organization or other reliable sources, would create the support base necessary to address the resistance to change. Although, interviewees also mentioned that ambassadorship or advocacy should come from a reliable and impartial source, not the provider directly.

“If people from that company are going to tell us that implementing their eMental solution makes you work faster, smarter, better, more fun… It's always going to be interpreted with a little bit of skepticism. So, if it's not available in the organizations themselves, it should be from a colleague, or a similar organization, that could help a lot. I think it's called ambassadorship”—Interviewee 11.

## Discussion

The present study described and evaluated the MiH, elaborating on its development process and the underlying expectations of implementation from the perspective of two stakeholder groups: employees within a commercial company and mental healthcare organizations. Both participant groups had an articulated definition of implementation, content- and structure-wise in line with core elements in the MiH. Elements of success and criticalities of MiH model and the underlying implementation process were identified. Main barriers were excessive workload, infrastructure and technical barriers, and ambiguity in the division of roles. Main strengths were high motivation of and good relationships between the stakeholders and customization of the offer or products.

The first aim of the present study was to describe the MiH and its conceptual underpinnings, including definitions of implementation of the participants involved. Participants in this study had clear, articulated, and shared definitions and expectations about what implementation entails. Particularly, implementation was defined by project managers in the mental healthcare organizations and implementation managers within Minddistrict as a complex process, with numerous stakeholders involved and steps to be followed. Interestingly, these elements are in line with the conceptualizations of implementation that are present in diffused academic frameworks [18, 19, 30]. Similarly, although the implementation managers in Minddistrict were not able to trace the origins of MiH to a specific academic reference, the model they developed still shares some properties with most implementation frameworks found in academic literature. For instance, MiH is divided into phases with a step-wise approach [18]. Secondly, the model has been described as flexible, adaptable, and iterative [18, 31]. Thirdly, similarly to the Process domain of the CFIR [18], it includes planning, engaging, executing and reflecting activities. However, the MiH model also presents several key differences from academic models. For instance, implementation frameworks such as the NASSS [19], CFIR [18] or Re-AIM [20] are evidence based. Furthermore, these models usually offer guidance for evaluation of implementation outcomes and processes, by offering tools and metrics to assess the success of the implementation. Also, they provide help in identifying barriers and facilitators of implementation, as well as strategies on to how to overcome them. Finally, they provide support for continuous implementation and offer a common language to understand implementation.

Holahan and colleagues [32] found that collective human perceptions have an impact on successful implementation of technology. Based on the results of this study, shared and complete definitions and expectations of implementation, in line with existing theory, might have contributed to successful implementation of eMental health technologies. Therefore, a possible improvement for (or addition to) the use of academic implementation frameworks could be the co-creation of a shared definition of implementation within the main stakeholders involved in the implementation process. In practice, developing a shared definition of implementation could shape the related expectations of what implementation should entail, therefore facilitating the goal setting process. Taking this one step further, setting clear, realistic, and concrete goals might provide organization with additional tools to determine the successfulness of the implementation process itself.

The second aim of the present study was to gain lessons learned from the development of MiH derived from its strengths and points of improvement. One of the main barriers to the implementation of Minddistrict that was identified through the present study was ambiguity in roles, which ultimately resulted in unclear responsibility and task division. This result is in accordance with Brantnell and colleagues [16], who concluded that diffusion of responsibility due to unclear role division might hinder implementation of innovations. Therefore, one of the main learning points derived by the results of this study is that, when considering adopting an implementation framework, a clear division of tasks should be ensured. Furthermore, fostering motivation of the stakeholders involved and addressing expectations could constitute a good practice in implementation processes according to our participants. In fact, the positive role of stakeholder engagement found in this study is in accordance with some of the most widely used implementation theories in implementation science [18, 31].

The results of the present study highlight the all-around importance of stakeholders, their attitude toward implementation, and their perspective. This might suggest that when planning for implementation, stakeholders should be actively involved throughout the process, e.g., in selecting implementation strategies, setting SMART [28] objectives, or creating and selecting implementation materials. This viewpoint is line with a participatory approach, that is common in eHealth and intervention development [12, 33, 34], but that could be further exploited and based on the findings of the present study beneficial to a better application of implementation frameworks. More specifically, this might imply the importance of a participatory approach towards planning, executing, and evaluating implementation processes.

A problematic consequence of implementation failure is research waste and the re-invention of the wheel caused by abandonment of newly developed innovations [12, 35, 36]. Often, this happens because of a poor fit with the intervention with the context it is implemented in [37]. This is also in line with the principles of Human-Centered Design (HCD) [38], advocating for the existence of a strong interrelationship between the design of an intervention, the needs of its users and the context in which it is used [37, 39]. In the present study, participants in mental healthcare valued the possibility of tailoring existing Minddistrict modules to their organizations’ specific needs and target groups. More specifically, although they expressed the desire to create their own modules, they often lacked time and resources to do so, and found in tailoring existing interventions a more viable alternative.

Another possible mitigation to implementation failure is (comprehensively and consistently) reporting, operationalizing, and measuring implementation strategies [40–42]. While some key tasks of the MiH show overlap with strategies suggested by influential works in the field [42], these are not operationalized or reported. To provide illustrative examples of similarities with the strategies suggested in the Expert Recommendations for Implementing Change (ERIC) [42], Minddistrict developed the MiH, which could constitute as a *formal implementation blueprint*. Furthermore, *trainings and educational meetings* are a vital part of the model. And finally, the *identification of leader-like* figures in the multidisciplinary team is another common strategy the MiH shares with the ERIC. The authors also suggests that cultivating partnership with academic institutions and researchers could “bring research skills to an implementation project” [42]. One practical example of this could be researcher providing commercial companies interested in implementing eHealth (or other innovations) with a stronger foundation in literature in building their models, and expertise on how to systematically report and operationalize implementation strategies that these models include.

**Fig. 2:**
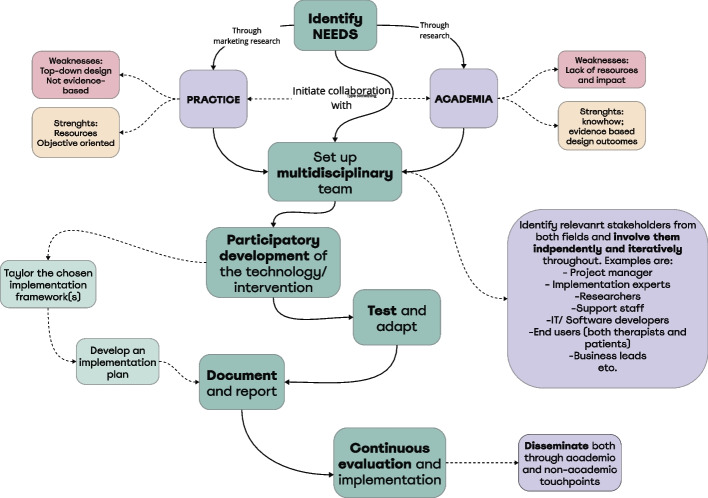
Flowchart of the proposed collaboration between academia and practice for the development and implementation of eHealth technologies

A strong collaboration between market-driven organizations and academia could also be a way to optimize implementation efforts and ensuring a better fit between humans, context, and technologies. More practically, companies such as Minddistrict could serve as a base for eHealth researchers to build their interventions, thus saving academic researchers platform design, building, and maintenance efforts, while also allowing a mainstream distribution of quality and evidence-based interventions to patients. Expanding on this, collaboration between academia and market should be set from the early stages of the development, not to keep research as a post-implementation activity. On the contrary: development, testing and implementation of eMental health keeps being top down (e.g., by not involving end-users and patients in the development process [31]. To ensure this is feasible however, new, and more viable research methods are required, focusing on iterative approaches, and promoting more agile evaluation golden standards. Figure 2 proposes an exemplificative flowchart on how optimal collaborations between academia and practice could take place, starting from the identification of a need in the real world, and counterbalancing each other’s complimentary strengths and weaknesses. This approach could ensure 1) a better fit between the technology being developed, its users and context and 2) taking implementation efforts into account already in the first stages of design.

Future studies should explore and describe strategies to effectively arrange fruitful collaborations between market and academia, throughout the whole design, development, and evaluation process in eMental health technologies, while also providing stakeholders with exhaustive knowledge on implementation science to manage expectations, foster engagement and create clear role division. Although an active feedback session with Minddistrict’s implementation team took place, future research should ideally follow the implementation process over a longer period of time, with continuous feedback iterations.

## Strengths and limitations

The participatory and multi-method design of the present explorative study is a strength. In fact, the research questions were developed in a combination of bottom-up and top-down approach, being informed by literature and previous research. Furthermore, the research questions were co-developed and validated by the Minddistrict implementation team to ensure a good fit between the research- and practical perspective. Finally, the interactive feedback session allowed us to conduct a member check and reflect on possible application of achieved results. Regarding limitations, this study only provides top-down points of view, because key-users such as therapists or patients were not involved in the study; this requires future research. Furthermore, Minddistrict implementation managers facilitated the recruitment of stakeholders in the implementing organizations. Although snowball sampling was also applied to reach more stakeholders, selection bias might have limited our recruitment, resulting in participants with mostly positive experiences with the intervention and/or implementation process.

## Conclusion

This study sheds light on the implementation of MiH, the implementation model developed by Minddistrict. The success of MiH's implementation process was facilitated by the presence of articulated and shared definitions of implementation among the stakeholders. Barriers such as role ambiguity and excessive workload were identified, highlighting the importance of clear role division and stakeholder engagement, not just when executing, but also planning for implementation. In fact, encouraging a synergetic, systematic, and consistent collaboration between market and academia in designing and implementing eHealth might be a mitigation strategy to the identified barriers. Academia and market should join their respective forces by, on one side, advocating for the use of evidence-based content and frameworks for the development and implementation of eHealth, and on the other hand by employing their practical know-how in goal setting and attention to contextual elements, but also by sharing access to their resources (e.g., facilitating platform development). In this light, stakeholders (e.g., therapists or other key-users) should be actively involved not only in the design phases, but also in the implementation phases. This way, not only evidence-based content would be more prominent, but research waste could also be limited, and stakeholders would take more agency and responsibility in implementation too.

### Supplementary Information

Supplementary Material 1.

Supplementary Material 2.

## Data Availability

The datasets generated and/or analyzed during the current study are not publicly available because it is not possible to fully anonymize the dataset but are available from the corresponding author on reasonable request.

## Abbreviations

MiH: Make it Happen
CFIR: Consolidated Framework for Implementation Research (18)
SMART goals: Specific, Measurable, Achievable, Relevant, and Time-bound (28)
NASSS: Non-Adoption, Abandonment, Scale-Up, Spread, And Sustainability (19)
ERIC: Expert Recommendations for Implementing Change (42)

## References

[1] Organization WH. Mental Health (2022) [cited 2023 25–09]. Available from: https://www.who.int/news-room/facts-in-pictures/detail/mental-health.

[2] Pyne JM, Patterson TL, Kaplan RM, Gillin JC, Koch WL, Grant I (1997). Assessment of the quality of life of patients with major depression. Psychiatric Services (Washington, DC).

[3] NIEUWS G. Prognose Van De Ontwikkeling Van De Arbeidsmarkt in De Ggz De Komende Tien Jaar [cited 2023 19–10]. Available from: https://www.oofggz.nl/nieuws/infographic-prognose-van-de-arbeidsmarkt-geestelijke-gezondheidszorg/.

[4] Bom J, Stockel J (2021). Is the grass greener on the other side? The health impact of providing informal care in the UK and the Netherlands. Soc Sci Med.

[5] Prevo L, Hajema K, Linssen E, Kremers S, Crutzen R, Schneider F (2018). Population characteristics and needs of informal caregivers associated with the risk of perceiving a high burden: a cross-sectional study. Inquiry.

[6] Lal S, Adair CE (2014). E-Mental health: a rapid review of the literature. Psychi Serv (Washington, DC).

[7] Kaonga NN, Morgan J (2019). Common themes and emerging trends for the use of technology to support mental health and psychosocial well-being in limited resource settings: a review of the literature. Psychiatry Res.

[8] Christensen H, Hickie IB (2010). E-Mental health: a new era in delivery of mental health services. Med J Aust.

[9] Cuijpers P, Donker T, van Straten A, Li J, Andersson G (2010). Is guided self-help as effective as face-to-face psychotherapy for depression and anxiety disorders? A systematic review and meta-analysis of comparative outcome studies. Psychol Med.

[10] Cuijpers P, Riper H, Andersson G (2015). Internet-based treatment of depression. Curr Opin Psychol.

[11] Andersson G, Hedman E (2013). Effectiveness of guided internet-based cognitive behavior therapy in regular clinical settings. Verhaltenstherapie.

[12] van Gemert-Pijnen L, Kelders SM, Kip H, Sanderman R (2018). Ehealth research, theory and development: a multi-disciplinary approach.

[13] Waltz TJ, Powell BJ, Matthieu MM, Damschroder LJ, Chinman MJ, Smith JL (2015). Use of concept mapping to characterize relationships among implementation strategies and assess their feasibility and importance: results from the Expert Recommendations for Implementing Change (Eric) study. Implement Sci.

[14] Ross J, Stevenson F, Lau R, Murray E (2016). Factors that influence the implementation of E-Health: a systematic review of systematic reviews (an Update). Implement Sci: IS.

[15] Varsi C, Ekstedt M, Gammon D, Ruland CM (2015). Using the consolidated framework for implementation research to identify barriers and facilitators for the implementation of an internet-based patient-provider communication service in five settings: a qualitative study. J Med Internet Res.

[16] Brantnell A. Exploitation of University-Based healthcare innovations: the behaviors of three key actors and influencing factors: Acta Universitatis Upsaliensis (2017).

[17] Bastoni S, Wrede C, da Silva MC, Sanderman R, Gaggioli A, Braakman-Jansen A (2021). Factors influencing implementation of Ehealth technologies to support informal dementia care: umbrella review. JMIR Aging.

[18] Damschroder LJ, Aron DC, Keith RE, Kirsh SR, Alexander JA, Lowery JC (2009). Fostering implementation of health services research findings into practice: a consolidated framework for advancing implementation science. Implement Sci: IS.

[19] Greenhalgh T, Wherton J, Papoutsi C, Lynch J, Hughes G, A'Court C (2017). Beyond adoption: a new framework for theorizing and evaluating nonadoption, abandonment, and challenges to the scale-up, spread, and sustainability of health and care technologies. J Med Internet Res.

[20] Glasgow RE, Vogt TM, Boles SM (1999). Evaluating the Public Health Impact of Health Promotion Interventions: The Re-Aim Framework. Am J Public Health.

[21] Heinsch M, Wyllie J, Carlson J, Wells H, Tickner C, Kay-Lambkin F (2021). Theories informing Ehealth implementation: systematic review and typology classification. J Med Internet Res.

[22] Nilsen P, Albers B, Shlonsky A, Mildon R (2020). Making Sense of Implementation Theories, Models, and Frameworks. Implementation Science 30.

[23] Birken SA, Powell BJ, Shea CM, Haines ER, Alexis Kirk M, Leeman J (2017). Criteria for selecting implementation science theories and frameworks: results from an international survey. Implement Sci: IS.

[24] Kouijzer MMTE, Kip H, Bouman YHA, Kelders SM (2023). Implementation of virtual reality in healthcare: a scoping review on the implementation process of virtual reality in various healthcare settings. Implement Sci Commun.

[25] Kip H, Buitelaar-Huijsse GKG, Kouijzer MTE, Kelders SM (2023). From theory to implementation in practice: a qualitative case study of the implementation of virtual reality in mental healthcare. Glob Implement Res Appl.

[26] Association AP. Qualitative Journal Article Reporting Standards (Jars-Qual) (2018) [cited 2024 28–3–2024]. Available from: https://apastyle.apa.org/jars/quantitative.

[27] Braun V, Clarke V (2023). Is thematic analysis used well in health psychology? A critical review of published research, with recommendations for quality practice and reporting. Health Psychol Rev.

[28] Doran GT (1981). There’s a smart way to write management’s goals and objectives. Manage Rev.

[29] Gureje O, Abdulmalik J, Kola L, Musa E, Yasamy MT, Adebayo K (2015). Integrating mental health into primary care in Nigeria: report of a demonstration project using the mental health gap action programme intervention guide. BMC Health Serv Res.

[30] Rogers E (2003). Diffusion of Innovations.

[31] van Gemert-Pijnen JE, Nijland N, van Limburg M, Ossebaard HC, Kelders SM, Eysenbach G (2011). A holistic framework to improve the uptake and impact of Ehealth technologies. J Med Internet Res.

[32] Holahan PJ, Aronson ZH, Jurkat MP, Schoorman FD (2004). Implementing computer technology: a multiorganizational test of Klein and Sorra’s model. J Eng Tech Manage.

[33] Yardley L, Ainsworth B, Arden-Close E, Muller I (2015). The person-based approach to enhancing the acceptability and feasibility of interventions. Pilot and Feasibility Studies.

[34] Bartholomew LK, Parcel GS, Kok G (1998). Intervention mapping: a process for developing theory- and evidence-based health education programs. Health Educ Behav.

[35] Breeman LD, Keesman M, Atsma DE, Chavannes NH, Janssen V, van Gemert-Pijnen L (2021). A multi-stakeholder approach to Ehealth development: promoting sustained healthy living among cardiovascular patients. Int J Med Informatics.

[36] Ivers NM, Grimshaw JM (2016). Reducing research waste with implementation laboratories. Lancet (London, England).

[37] Pieterse M, Kip H, Cruz-Martínez RR. The complexity of Ehealth implementation: a theoretical and practical perspective. *eHealth Research, Theory and Development: A Multi-Disciplinary Approach*. London: Routledge 2018:247–70.

[38] Burns C (2018). Human-centred design. Ehealth research, theory and development.

[39] Kip H. The added value of Ehealth: improving the development, implementation and evaluation of technology in treatment of offenders. 2021.

[40] Proctor E, Silmere H, Raghavan R, Hovmand P, Aarons G, Bunger A (2011). Outcomes for implementation research: conceptual distinctions, measurement challenges, and research agenda. Admin Policy Mental Health Mental Health Serv Res.

[41] Rudd BN, Davis M, Beidas RS (2020). Integrating implementation science in clinical research to maximize public health impact: a call for the reporting and alignment of implementation strategy use with implementation outcomes in clinical research. Implement Sci: IS.

[42] Powell BJ, Waltz TJ, Chinman MJ, Damschroder LJ, Smith JL, Matthieu MM (2015). A refined compilation of implementation strategies: results from the Expert Recommendations for Implementing Change (Eric) Project. Implement Sci.

