# Supplementary material for: From theory to practice in implementation science: qualitative insights from the implementation model developed by a commercial eMental Health provider
Source: Implement Sci Commun. 2024 Jul 4;5:72. doi: 10.1186/s43058-024-00610-y (PMC11225237; doi:10.1186/s43058-024-00610-y)
Supplement: Supplementary file 1 — Supplementary Material 1. [file 43058_2024_610_MOESM1_ESM.docx]

**Additional file 1- Interviews and focus group guides**

**Interview guide 1: Definition of implementation- Minddistrict Implementation Managers**

| Greet the interviewee, collect informed consent form, start the recording.   1. Please take up to 5 minutes and think of how you would explain what implementation is to someone who does not know anything about it. Pretend you are writing the “implementation” section in a dictionary or sketching a concept map. Feel free to sketch or draw or write (Please do not use google). 2. What is implementation to you? Please read/ tell your definition 3. How did you come to this definition? 4. What are the steps to implementation? (When does it begin/end?) 5. Who is involved and how? (What are their tasks?) 6. Please comment on the role of Users/Context inner-outer/technology role in implementation (using your map/drawing)   Thank the interviewee, collect drawing/scheme. |
| --- |

**Focus group guide: Description of MiH with Minddistrict Implementation Managers**

| Question | Prompts/ checklist for main desired points |
| --- | --- |
| Phase 1 | |
| CFIR DOMAIN: Process | |
| 1)Let’s talk about the first phase, what would be a good English name for it?  1B) Can you **describe the first (second, etc) phase of** the plan for implementing Minddistrict?  1C) Please drag and drop any relevant resource  *45 min (9 mins per phase)* | Prompts (x5 phases) *How detailed is the plan?*  *Is the plan overly complex? Understandable? Realistic and feasible?*  *Who knows about it?*  *What is your role in the planning process?*  *Who (else) is involved in the planning process?*  *What are their roles?*  *Are the appropriate people involved in the planning process?*  *How engaged are they?*  *What if you have to modify or revise your plan due to barrier, errors, or mistakes?* |
| What are the main problems or points of improvements of this model? *5 min* |  |
| Phase 2 | |
| 2) Once Minddistrrict is implemented within an organization, do you assess implementation outcomes?  *3 min* | *What kind of information do you collect?* |
| CFIR DOMAIN: Outer setting | |
| 3) Who are the end users of MD?  3b) How are they involved throughout the phases? *5 min* |  |
| 4) To what extent were the needs and preferences of the end users by your organization considered when deciding to implement Minddistrict?  4b) Can you describe specific examples?  *3 min* | *How well do you think the intervention will meet the needs of the individuals served by your organization?* |
| 5a) What are policies, regulations, or guidelines that you need to account for when implementing Minddistrict?  5b) What kind of financial or other incentives could influence the decision to implement the Minddistrict? *4 min* |  |
| 6) Think about all stakeholders of MD, who are they and what is their role throughout the phases? *5 min* | Get some kind of customer/stakeholder journey.  List of professionals:  -  -  -  -  - |
| 7) About the plan: When was it developed? How? Describe the process in detail. Since when it has been used consistently? *4 min* |  |

| Prompt questions: To be asked in reaction to specific topics participants might raise (if there’s time) | |
| --- | --- |
| CFIR DOMAIN: Process | |
| GOAL SETTING PHASE: - How you support organizations in setting goals for Implementation? Do you set goals yourself? *3 min* | How will goals be communicated in the organization? To whom will they be communicated?  What are the goals? How and to whom will they be communicated? |
| IF THEY MENTION AMBASSADORS: - Other than the formal implementation leader, are there people in your organization who are likely to champion (go above and beyond what might be expected) the intervention?  - What kinds of behaviors or actions do you think this individual/champion will exhibit? *3 min* | Were they formally appointed in this position, or was it an informal role?  What position do these champions have in your organization?  How do you think they will help with implementation? Getting people to use the intervention? |
| Closing: Ask for help with recruitment; great and thank the participants | |

Focus Group Miro Boards:


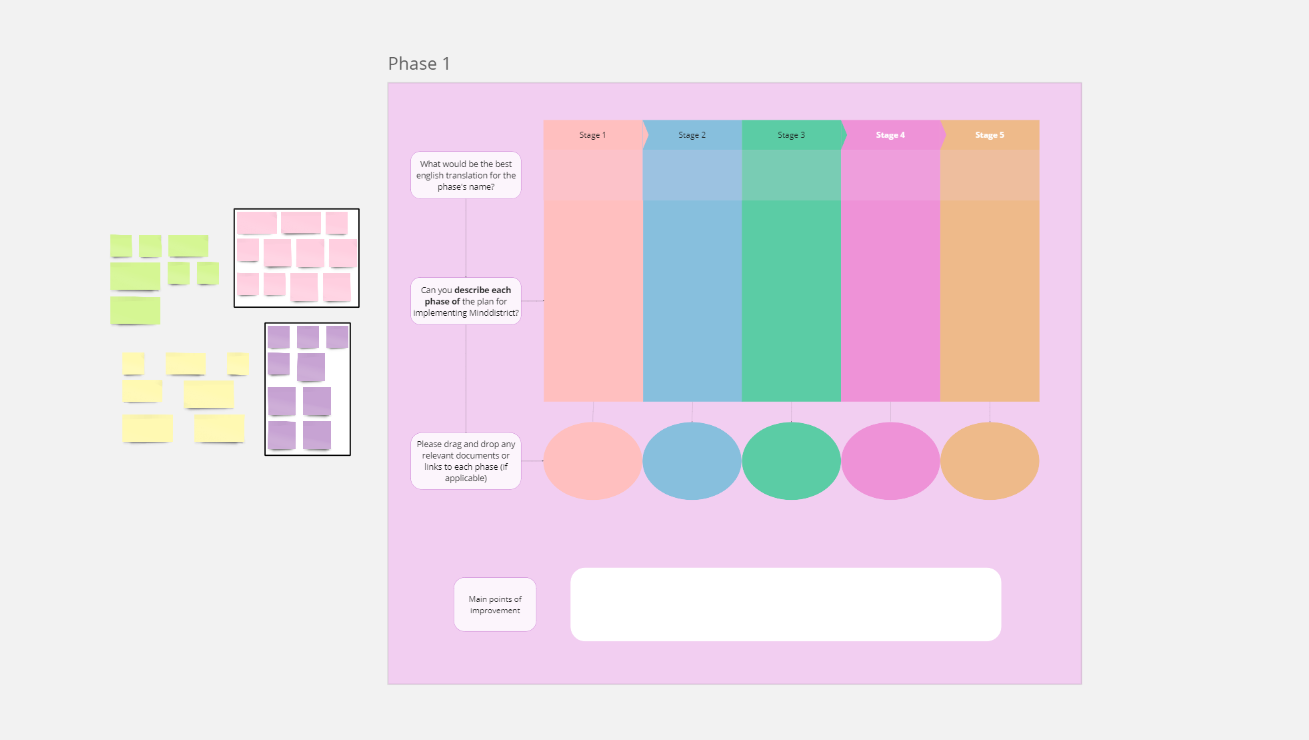


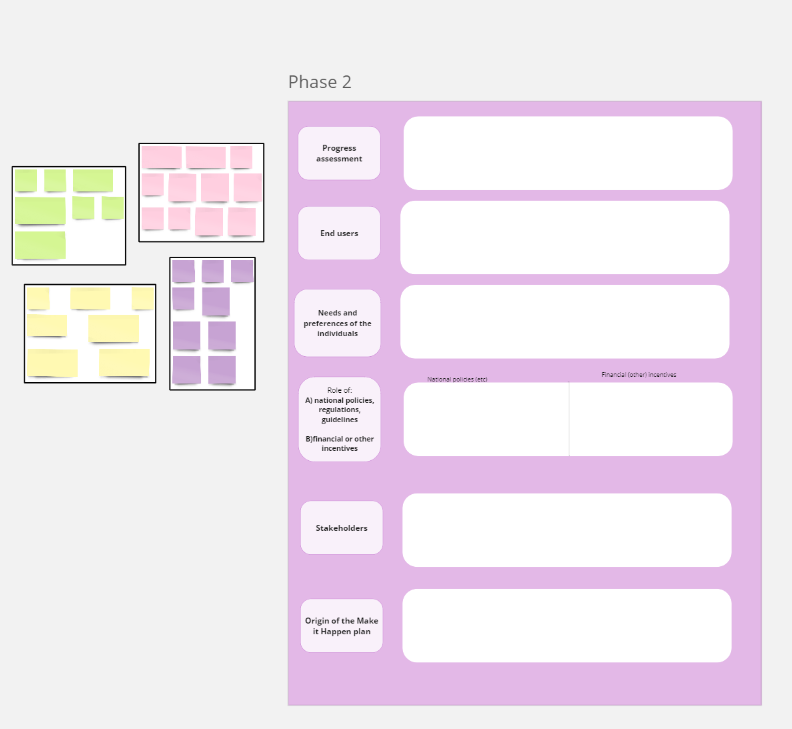


**Interview guide 3: stakeholder experience with the implementation model of Minddistrict (Dutch below)**

| 1. What is your role in your organization? 2. What is implementation of eHealth for you and your organization?   Prompt(s): Who is responsible for it? were your expectations matched in the experience with Minddistrict?   1. In what year did you take part to the implementation of Minddistrict? 2. In a few words, what is your general memory/feeling about it?   *Thank you. Now we are going to move on to questions about your experience with specific activities and phases of the implementation process with Minddistrict. For this section, we have 7 questions with 25 minutes, so we’ll try to fit in that time.*  ***Present them with a slide to visualize the different phases of MiH***   1. Before we begin, are you familiar with this structure in your experience? (If not, go over briefly and reassure them it’s not a problem.) 2. How would you describe your experience with the onboarding in Minddistrict? What were strength and pitfalls? 3. How would you describe your experience with setting goals for your organization with Minddistrict? What were strength and pitfalls? 4. How would you describe your experience with creating a patient/therapist journey for Minddistrict? What were strength and pitfalls? 5. How would you describe your experience with the customizing and building the content for the catalogue (building the welcome module)? What were strength and pitfalls? 6. (For therapists) How would you describe the trainings? What were strength and pitfalls?   10b (For non therapists): Have you gotten any feedback about the trainings from the therapists in your organization? What were strengths and pitfalls?   1. How would you describe your experience with the evaluation process? What were strength and pitfalls?   11b: if you had experience with the ehealth Check up tool: How would you describe your experience with the eHealth check-up tool? What were strength and pitfalls?  Thank you we are almost through with the questions. To close up:   1. Do you remember what happened after evaluation?   Prompt: Were your expectation matched?   1. What were points of improvement in general for the implementation process of Minddistrict? 2. Bonus: were you/ your colleagues or your patients involved in the design of modules? How?   Thank the interviewee for their time and close the interview. |
| --- |

| Interviewschema: evaluatie van belanghebbenden van het implementatiemodel van Minddistrict  *Dank de geïnterviewde, stel uzelf en het project voor, begin met het opnemen en zorg ervoor dat ze hiermee akkoord gaan.*  Tijdens dit interview zal ik u vragen stellen over uw ervaring met het implementatieproces van Minddistrict. Het algemene doel van dit project is inzicht te krijgen in punten waarop Minddistrict hun implementatiemodel kan verbeteren.  Om te beginnen:   1. Wat is jouw rol binnen je organisatie? 2. Wat betekent implementatie voor jou en je organisatie? Wie is er verantwoordelijk voor?   Prompt: Kwamen je verwachtingen (over implementatie) overeen met je ervaringen met Minddistrict?  In welk jaar heb je deelgenomen aan de implementatie van Minddistrict?   1. Kun je kort beschrijven wat je algemene herinnering/gevoelens hierover zijn?   Dankjewel. We gaan nu verder met vragen over je ervaringen met specifieke activiteiten en fasen van het implementatieproces met Minddistrict. Voor dit gedeelte hebben we 7 vragen en 25 minuten, dus we zullen proberen dit binnen die tijd te behandelen.  ***Laat de dia zien***   1. Voordat we beginnen, ben je bekend met deze structuur op basis van je ervaring? (Indien niet, geef een korte uitleg en verzeker hen dat het geen probleem is.) 2. Hoe zou je je ervaring met de onboarding bij Minddistrict omschrijven? Wat waren sterke punten en valkuilen? 3. Hoe zou je je ervaring met het stellen van doelen voor je organisatie met Minddistrict omschrijven? Wat waren sterke punten en valkuilen? 4. Hoe zou u uw ervaring met het creëren van de cliëntreis en workflow/werkproces voor Minddistrict omschrijven? 5. Hoe zou je je ervaring met het aanpassen en opbouwen van de inhoud voor de catalogus (het opbouwen van het welkomstmodule) bij Minddistrict omschrijven? Wat waren sterke punten en valkuilen? 6. (Voor therapeuten) Hoe zou je de trainingen omschrijven? Wat waren sterke punten en valkuilen?   10b. (Voor niet-therapeuten) Heb je feedback gekregen over de trainingen van therapeuten binnen je organisatie? Wat waren sterke punten en valkuilen?   1. Hoe zou je je ervaring met het evaluatieproces omschrijven? Wat waren sterke punten en valkuilen?   11b. Als je ervaring hebt met de eHealth Check up-tool: hoe zou je je ervaring met de eHealth Check up-tool omschrijven? Wat waren sterke punten en valkuilen?  Dankjewel, we zijn bijna klaar met de vragen. Ter afsluiting:   1. *Weet je nog wat er na de evaluatie is gebeurd?*   *Prompt: Kwam dit overeen met je verwachtingen?*   1. *Wat waren in het algemeen punten ter verbetering voor het implementatieproces van Minddistrict?* 2. *Bonus: Was jij, je collega's of je patiënten betrokken in het maken van de modules? Op welke manier?* |
| --- |
