# Supplementary material for: From theory to practice in implementation science: qualitative insights from the implementation model developed by a commercial eMental Health provider
Source: Implement Sci Commun. 2024 Jul 4;5:72. doi: 10.1186/s43058-024-00610-y (PMC11225237; doi:10.1186/s43058-024-00610-y)
Supplement: Supplementary file 2 — Supplementary Material 2. [file 43058_2024_610_MOESM2_ESM.docx]

**Additional File 2: Overview of Codes**

| **CODE** | **THEME** | **SUBTHEME** | Number of Times coded |
| --- | --- | --- | --- |
| Mental model of Implementation | This theme relates to all fragments describing the interviewee mental model of Implementation |  | 12 |
| Strengths | This theme relates to all fragments highlighting a positive aspect of the MiH |  | 43 |
|  |  | Relationships:  This code relates to all the positive aspects of the MiH that have to do with experiencing positive relationships with the implementation managers | 8 |
|  |  | Organization: This code relates to practical aspects in the method that relate to the organization and overview of the tasks given by Minddistrict | 29 |
|  |  | Motivation: This code relates to aspect that are related to the motivation of the stakeholders involved | 6 |
|  |  | Goal setting: This code relates to all positive aspects/ positive comments about the goal setting phase | 8 |
|  |  | Customization: This code relates to the possibility of personalizing content to the implementing organizations | 4 |
|  |  | Training | 5 |
| Barriers | This theme relates to all fragments highlighting a negative aspect of the MiH or the implementation trajectory |  | 69 |
|  |  | Workload | 12 |
|  |  | Choice of modules | 4 |
|  |  | Practical Barriers | 10 |
|  |  | Heterogeneity | 2 |
|  |  | Unclear division of roles | 12 |
| Possible Improvements | This theme relates to all fragments suggesting a possible improvement for the MiH |  | 34 |
|  |  | Ambassadorship |  |
|  |  | More time and information | 10 |
|  |  | Personalization | 4 |
|  |  | Choice of modules | 3 |

Disclaimer:

Codes like “choice of modules” were both present as in the theme “barriers” and “possible improvements” depending on the context in which the interviewees were mentioning it in.
